# Effects of Zn–ZnO Core–Shell Nanoparticles on Antimicrobial Mechanisms and Immune Cell Activation

**DOI:** 10.1021/acsanm.3c03241

**Published:** 2023-09-11

**Authors:** Luísa Fialho, Augusto Costa-Barbosa, Paula Sampaio, Sandra Carvalho

**Affiliations:** † CEMMPRE, Departamento de Engenharia Mecânica, Universidade de Coimbra, 3030-788 Coimbra, Portugal; ‡ CBMA, Departamento de Biologia, Campus de Gualtar, Universidade do Minho, 4710-057 Braga, Portugal; § IPN − LED & MAT − Instituto Pedro Nunes, Rua Pedro Nunes, 3030-199 Coimbra, Portugal

**Keywords:** antimicrobial mechanisms, surface modification, plasma electrolytic oxidation, magnetron sputtering, dental implants

## Abstract

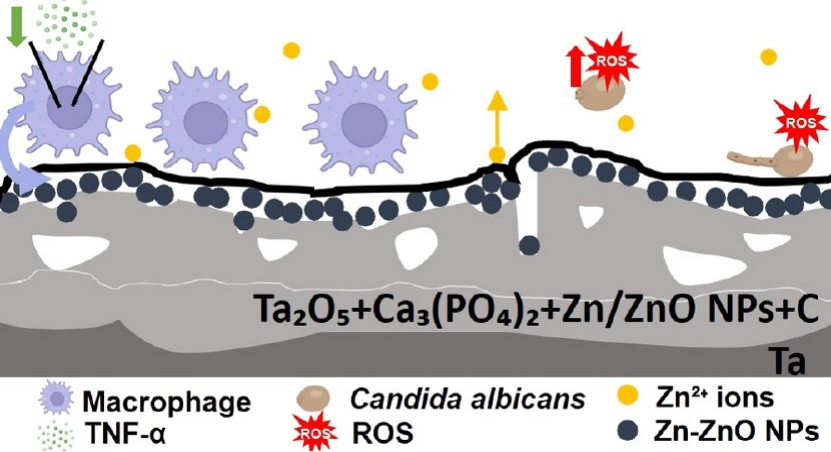

The deposition of zinc–zinc oxide nanoparticles (Zn–ZnO NPs) onto porous Ta_2_O_5_ surfaces enriched with calcium phosphate by DC magnetron sputtering was investigated to improve the surface antimicrobial activity without triggering an inflammatory response. Different sizes and amounts of Zn NPs obtained by two optimized different depositions and an additional thin carbon (C) layer deposited over the NPs were explored. The deposition of the Zn NPs and the C layer mitigates the surface porosity, increasing the surface hydrophobicity and decreasing the surface roughness. The possible antimicrobial effect and immune system activation of Zn–ZnO NPs were investigated in *Candida albicans* and macrophage cells, respectively. It was found that the developed surfaces displayed a fungistatic behavior, as they impair the growth of *C. albicans* between 5 and 24 h of culture. This behavior was more evident on the surfaces with bigger NPs and the highest amounts of Zn. The same trend was observed in both reactive oxygen species (ROS) generation and loss of *C. albicans*’ membrane integrity. After 24 h of culture, cell toxicity was also dependent on the amount of the NPs. Cell toxicity was observed in surfaces with the highest amount of Zn NPs and with the C layer, while cells were able to grow without any signs of cytotoxicity in the porous surfaces with the lowest amount of NPs. The same Zn-dose-dependent behavior was noticed in the TNF-α production. The Zn-containing surfaces show a vastly inferior cytokine secretion than the lipopolysaccharide (LPS)-stimulated cells, indicating that the modified surfaces do not induce an inflammatory response from macrophage cells. This study provides insights for understanding the Zn amount threshold that allows a simultaneous inhibition of the fungi growth with no toxic effect and the main antimicrobial mechanisms of Zn–ZnO NPs, contributing to future clinical applications.

## 1 Introduction

Dental implants are a common therapeutic process to treat edentulous patients. After the dental implant insertion, inflammation is induced in the implant site.^1,2^ The local inflammation can alter the immunological state, making the implant more susceptible to microbial colonization.^1^ During the period that the gap between the implant surface and host bone is filled by bone ingrowth (osteogenesis), there is a probability of the microorganisms entering into this gap and adhering to the implant surface.^3^ This microbiota (bacteria, fungi) from the mucosal flora^4^ can induce the appearance of infections and consequent complications,^5,6^ such as peri-implantitis.^7,8^ Peri-implantitis is currently qualified as an emerging public health problem without an effective and predictive treatment.^9^

The initial biological response to a biomaterial is modulated by the immune system,^10^ composed of macrophages. Macrophages play two crucial roles as they are responsible for inflammation and bone healing regulation^11^ and avoid infection.^12^ Macrophage’s role and how it relates to peri-implantitis infection is extremely vital for the long-term maintenance of dental implants.^12^ Macrophage activation can be modulated by the implant surface properties,^10,13^ affecting the healing process and long-term implant stability.^10^ Thus, this initial cellular response is a determinant of dental implant success.^10^ However, only 10% of the literature is dedicated to immune cell interactions (including macrophages or monocytes), while over 90% of the research is focused on osteoblast and fibroblast behavior to the material.^12^ Less information is available concerning the response of macrophages to implanted biomaterials.

The surface modification of dental implants has been widely investigated to increase the surface roughness and wettability, as well as change the chemical composition by incorporation of hydroxyapatite (calcium phosphates) to improve surface bioactivity and further decrease the healing time and leading to the implant’s long-term success.

Tantalum (Ta) has been investigated as a dental implant material, being well documented as biocompatible and bioactive,^14^ leading to a strong bonelike apatite layer formation.^5^ Also, zinc (Zn) has been explored as it simultaneously displays osteogenic ability and antimicrobial effect against Gram-negative and Gram-positive bacteria as well as fungi, such as *Escherichia coli*, *Staphylococcus aureus*, and *Candida albicans*, respectively.^15,16^ Also, zinc oxide (ZnO) nanoparticles (NPs) play a dual role in antimicrobial and immunomodulatory activities.^17^ The precise antimicrobial mechanism is still under debate, although the reactive oxygen species (ROS) generation and zinc ion release are the most proposed and accepted ones. ZnO NPs internalization can cause membrane disruption and dysfunction.^18^ Additionally, the potential use of ZnO NPs as antimicrobial agents has been investigated not only for biomedical devices^19^ but also for water remediation^20,21^ and photocatalysis.^22^

In our previous studies, we reported for the first time the novel approach of modified Ta surfaces for dental implants. The Ta surface was modified by plasma electrolytic oxidation (PEO), developing a micro/nanoporous tantalum oxide (Ta_2_O_5_) layer enriched with calcium phosphate (CaP),^23^ and Zn–ZnO NPs were deposited over the porous Ta_2_O_5_ by DC magnetron sputtering and covered by a thin carbon (C) layer.^14^ Although the porous Ta_2_O_5_ doped with Zn–ZnO NPs covered by the C layer significantly improved the initial osteoblastic cell adhesion and proliferation,^14,23^ the modified Ta surfaces did not show a significant inhibition on bacterial growth.^14^

As one step further for the prevention of dental implant-associated infections without an inflammatory response, in this work, the deposition of Zn–ZnO NPs was optimized to achieve smaller NPs/lower amount and larger NPs/higher amount, with and without the protective thin C layer. The structural and chemical properties of the samples were evaluated, as well as the antifungal activity and macrophages’ activation induced by the modified Ta surfaces.

## 2 Experimental Section

### 2.1 Surface Biofunctionalization

Porous Ta_2_O_5_ surfaces enriched with CaP were obtained by PEO treatment of Ta surfaces (Ta, 99,95% purity, Testbourne), using an electrolyte composed of the precursors of calcium and phosphorous, 0.35 M calcium acetate (C_4_H_6_CaO_4_, 99%, Biochem, Chemopharma) and 0.12 M β-glycerol phosphate ((HOCH_2_)_2_CHOP(O)(ONa)_2_·*x*H_2_O, 98%, Sigma-Aldrich), under 200 V for 30 min. The resulting surfaces were named TaCaP.

Then, Zn NPs were deposited over the TaCaP surfaces and ultrathin carbon TEM grids by DC magnetron sputtering using two deposition conditions (named TaCaP-Zn1 and TaCaP-Zn2 surfaces). Then, the Zn NPs were covered by a thin C layer (called TaCaP-Zn1C and TaCaP-Zn2C surfaces), which resulted from the dissociation of the acetylene gas (C_2_H_2_). The deposition conditions are displayed in Table 1. The detailed Zn NPs deposition is reported in ref (24).

**Table 1:** Deposition Conditions of TaCaP-Zn1, TaCaP-Zn2, TaCaP-Zn1C, and TaCaP-Zn2C Surfaces

	Zn NP deposition				C layer deposition					
samples	pressure (Pa)	current density (mA/cm^2^)	time (s)	Ar flow (sccm)	pressure (Pa)	current (A)	frequency (Hz)	time (s)	C_2_H_2_ flow (sccm)	Ar flow (sccm)
TaCaP-Zn1	6.3	0.5	1000	80						
TaCaP-Zn2	2.0	1	500	80						
TaCaP-Zn1C	6.3	0.5	1000	80	3	0.4	200	60	20	80
TaCaP-Zn2C	2.0	1	500	80	3	0.4	200	60	20	80

**Figure 1:**
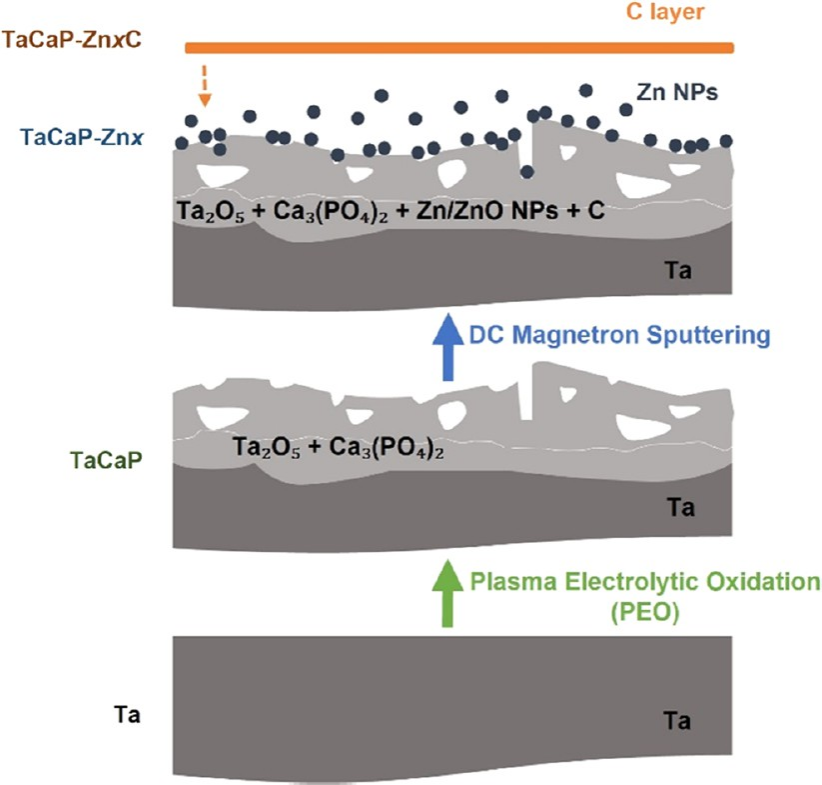
Schematic diagram of the Ta biofunctionalization process by both PEO and DC magnetron sputtering surface treatments and structure of TaCaP, TaCaP-Zn*x*, and TaCaP-Zn*x*C samples (*x* = 1, 2).

Figure 1 displays the experimental process of Ta biofunctionalization by both PEO and DC magnetron sputtering surface treatments.

### 2.2 Surface Characterization

The surfaces’ morphology was observed by scanning electron microscopy (SEM) with a NanoSEM 200 microscope (FEI), at 10 kV in secondary electron mode. The Zn NPs deposited onto the C lacey TEM grids were evaluated by scanning transmission electron microscopy (STEM, Nova NanoSEM 200 microscope, FEI) to evaluate their morphology. The elemental chemical composition of the surfaces was determined by X-ray photoelectron spectroscopy (XPS), using a Kratos AXIS Ultra HSA, with a monochromatic Al Kα X-ray source (1486.7 eV). The electric charge effect was corrected by the reference to the carbon peak (285 eV). The surfaces’ topography was evaluated by atomic force microscopy (AFM, CSI – Nano-Observer Atomic Force Microscope) in tapping mode. AFM micrographs were taken over scanning areas of 10 × 10 μm^2^, and a 3D profile was generated. The mean roughness (Sa) was obtained through the analysis of the AFM micrographs (scanning areas of 2 × 2 μm^2^) by Gwydion from three independent measurements. The surface wettability was determined by the sessile drop test, using an OCA20 Plus optical contact angle measuring system (DataPhysics, Germany). A droplet of 2 μL of Milli-Q ultrapure water was suspended on each surface with a dosing rate of 2 μL/s at room temperature (RT). Water contact angle measurements were performed in triplicate per surface.

### 2.3 Antimicrobial Activity

#### 2.3.1 Microbial Cell Culture

The samples were sterilized in dry heat (180 °C) for 2 h, and a preinoculum (preculture) of *C. albicans* SC 5314 cells^25^ was prepared by picking one colony and inoculating in 10 mL of yeast peptone dextrose (YPD) growth medium (1% w/v yeast extract, 1% w/v peptone, and 2% w/v glucose, Formedium). The preinoculum was incubated at 30 °C with 200 rpm in orbital agitation overnight (Benchtop Shaking Incubator 222DS). After the growth of the preinoculum, *C. albicans* SC 5314 cells were transferred to a new culture tube, at an optical density (O.D._600 nm_) of 0.2, with 20 mL of YPD. This inoculum (cell culture) was incubated in 200 rpm orbital agitation at 30 °C, and the cell growth was monitored until reaching an O.D._600 nm_ of 0.5 (corresponding to the *C. albicans* cells’ exponential growth phase). At this point, 200 μL of the inoculum was added to the sterilized sample surfaces and incubated at 30 °C for 5 and 24 h (cell viability time points).

#### 2.3.2 Microbial Viability

To determine the colony counts (CFU/mL), a serial dilution (from 10^–1^ to 10^–5^) of *C. albicans* cultures was performed for each time point. For the first dilution (10^–1^), 20 μL of the cell culture on each surface sample was diluted in 180 μL of deionized water, from where, after resuspension, 50 μL was pipetted to 450 μL of deionized water (second dilution – 10^–2^), and so on. After all the dilutions were prepared, 50 μL of the latest three dilutions were pipetted five times onto YPD agar plates. The TaCaP sample was used as a control. The experiment was performed in quadruplicate for each sample group.

After 5 and 24 h of cultures, the samples were observed by SEM (Nova NanoSEM 200 microscope, FEI) at 10 kV. Samples were coated with an 8 nm Au/Pd thin film. Before SEM analysis, samples were fixed in 2.5% (v/v) glutaraldehyde (diluted in PBS) and dehydrated in graded ethanol solutions (50, 70, 90, and 100% v/v) and hexamethyldisilane/ethanol (50, 70, 90, and 100% v/v, HMDS, Sigma-Aldrich) series.

The generation of reactive oxygen species (ROS) and the cellular membrane integrity of *C. albicans* were evaluated by flow cytometry (Cytoflex System B4-R2-V0, Beckman Coulter) after 5 and 24 h of interaction with the Zn-containing surfaces, the positive control group (TaCaP) and the negative control (heat-killed cells). At each time point, the cells were incubated with Sytox Green (membrane integrity marker, final concentration of 50 μM) for 10 min and dihydroethidium (DHE) (ROS marker, final concentration of 10 μM) for 5 min in the dark. At least 30 000 events were analyzed per sample. The experiment was performed in quadruplicate for each sample group. The negative control consists of marked cells cultured on the TaCaP surface. A forward-scattered (FSC) and side-scattered (SSC) quadrant threshold was set to exclude cell debris. All the events, except debris, were investigated.

### 2.4 Immune System Activation

#### 2.4.1 Cell Culture

The immune system activation was evaluated using the macrophage cell line J774A.1. Macrophage cells were cultured in Dulbecco’s Modified Eagle’s Medium (DMEM, with glucose, glutamine, and HEPES, Gibco, UK) supplemented with 1 mM pyruvic acid sodium salt (Merck, Germany) and 10% (v/v) of heat-inactivated fetal bovine serum (FBS, Gibco, U.K.). The incubation was carried out at 37 °C in a humidified atmosphere with 5% CO_2_.

TaCaP sample and Zn-containing surfaces were sterilized in dry heat (180 °C) for 2 h and then plated onto 6-well plates. First, the samples were incubated with 500 μL of complete medium for 30 min at 37 °C and 5% CO_2_. Then, the cellular suspension was seeded at 5 × 10^5^ cells/well and incubated for 24 h at 37 °C with a humidified atmosphere of 5% CO_2_.

#### 2.4.2 Cell Viability

The MTT assay was used to assess cellular metabolic activity. After 24 h of incubation, the samples were transferred to another 6-well plate, and 2.5 mL/well of complete DMEM with MTT (final concentration of 0.5 mg/mL, Sigma) was added. The samples were incubated for 2 h at 37 °C with an atmosphere of 5% CO_2_. After that, the supernatant was discarded, and the insoluble formazan crystals were solubilized with 2.5 mL/well of DMSO/ethanol (1:1). Then, 100 μL/well of the MTT formazan solution was collected and plated onto 96-well plates (VWR), and the absorbance measured at 570 nm and RT on a SpectraMax Plus 384 microplate reader (Molecular Devices). Macrophage cells grown on a 6-well plate without any sample were also tested as a reference. The cell viability was normalized by the sample’s area and well’s area (positive control – untreated cells).

#### 2.4.3 Cell Morphology

Following 24 h of incubation, the cell culture medium was discarded, and cells were washed in phosphate buffer solution (PBS) three times. Then, the cells were fixed with 2.5% (v/v) glutaraldehyde (diluted in PBS) and dehydrated in graded ethanol solutions (50, 70, 90, and 100% v/v) and hexamethyldisilane/ethanol (50, 70, 90, and 100% v/v, HMDS, Sigma-Aldrich) series. Finally, the cells were sputter-coated with an 8 nm thick Au/Pd film and observed by SEM (Nova NanoSEM 200 microscope, FEI) at 10 kV in SE mode.

#### 2.4.4 Cytokine Production

The enzyme-linked immunosorbent assay (ELISA) was performed to quantify TNF-α in the cell culture supernatant. For that, after 24 h of incubation, 1 mL/well of cell culture supernatant was collected and stored at −20 °C for further analysis. The proinflammatory cytokine TNF-α was quantified using a Mouse TNF-α uncoated ELISA kit (Invitrogen), following the manufacturer’s instructions, and the absorbance was measured at 450 and 570 nm at RT on an Infinite M200 NanoQuant microplate reader (Tecan, EUA). Cells treated with polysaccharides (LPS) were used as a positive control of inflammation, as described in ref (26), while untreated cells were used as a negative control of inflammation.

### 2.5 Statistical Analysis

Statistically significant differences between the sample groups were measured using one-way ANOVA and Tukey’s multiple comparison tests to assess the significance of the effects of the exposure concentration and duration and their interaction.

All statistical analyses were carried out using the GraphPad Prism8 statistical software package. All the data are expressed as mean ± standard deviation.

## 3 Results and Discussion

### 3.1 Morphological, Chemical, and Physical Surface Properties

**Figure 2:**
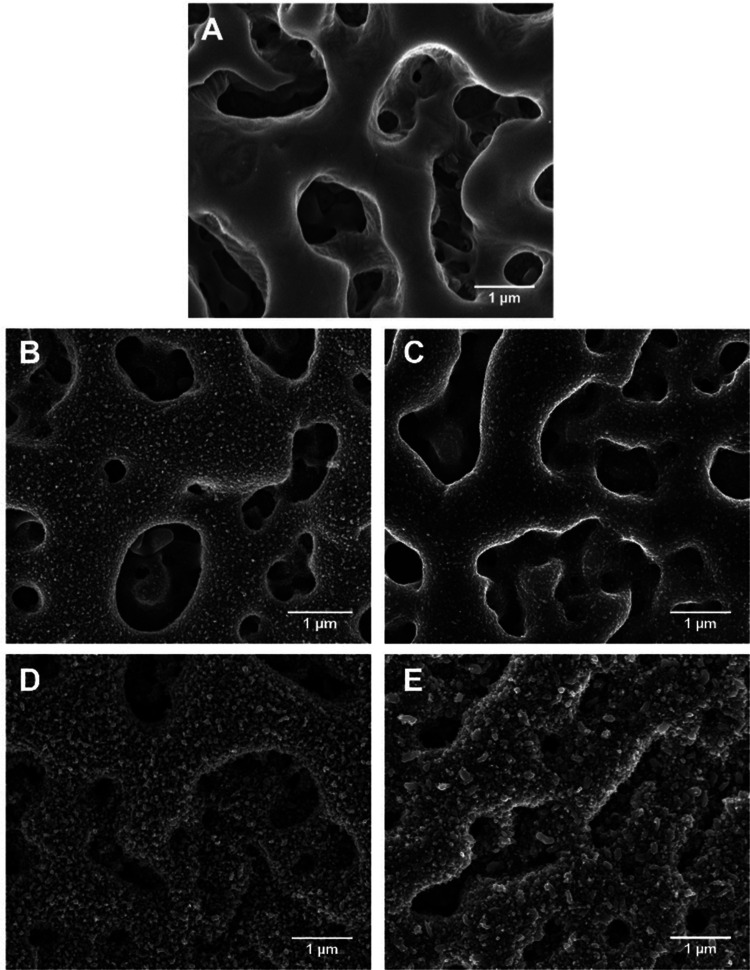
SEM micrographs of the modified Ta surfaces: (A) TaCaP; (B) TaCaP-Zn1; (C) TaCaP-Zn1C; (D) TaCaP-Zn2; and (E) TaCaP-Zn2C samples. Scale bar: 1 μm.

The TaCaP surface, resulting from the PEO treatment, shows a micro/nanoporosity with different diameters and distributions (Figure 2A), in agreement with the previously reported results.^14^ The deposition of Zn NPs over the TaCaP surface was performed by the DC magnetron sputtering technique under different conditions (displayed in Table 1). The first deposition condition leads to the formation of small NPs well distributed over the surface and inside the porosities (Figure 2B), while the second condition results in the deposition of larger Zn NPs that cover the smaller pores (Figure 2D). The deposition of the thin C layer over both Zn NPs scenarios does not result in significant changes in the corresponding surfaces’ morphology (Figure 2C,E).

**Figure 3:**
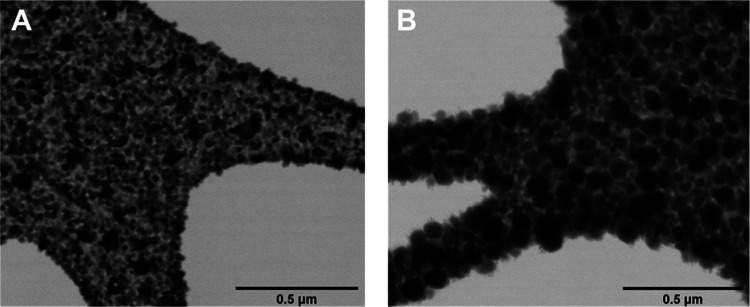
STEM micrographs of the Zn NP deposition conditions of (A) TaCaP-Zn1 and (B) TaCaP-Zn2 samples over carbon lacey grids. Scale bar: 0.5 μm.

To better illustrate the NPs morphologies, the Zn NPs were also deposited under the same conditions (Table 1) over carbon lacey grids (Figure 3). As aforementioned, the first condition (TaCaP-Zn1 sample – Table 1) deposits small NPs with different sizes and shapes (Figure 3A). To increase the amount of Zn NPs on the TaCaP surface (TaCaP-Zn2 sample – Table 1), the target current density was increased twice to increase the deposition rate,^27,28^ while the working pressure was decreased to half to produce more energetic species^29,30^ and increase the adatom mobility.^30^ The deposition time was decreased to avoid the formation of a continuous Zn thin film. These deposition parameters, as expected, result in significantly larger Zn NPs (Figure 3B).

To assess the chemical composition and confirm the Zn on the top surface, the obtained samples were characterized by XPS.

**Figure 4:**
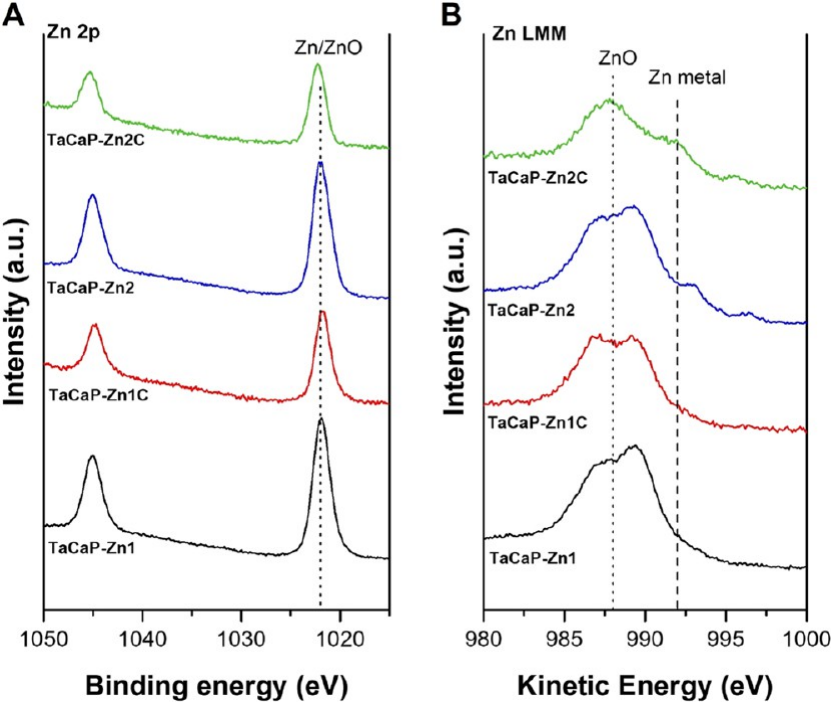
(A) XPS spectra of Zn 2p of the Zn-containing surfaces and (B) Zn LMM Auger peak.

The Zn NPs were effectively deposited over the surface by DC magnetron sputtering (Figure 4). As expected, when the deposition conditions are changed to increase the amounts of NPs, the Zn spectra shift. The XPS spectra (Figure 4A) of the surfaces for Zn 2p display a doublet at around 1022 and 1045 eV, corresponding to Zn 2p_3/2_ and Zn 2p_1/2_, respectively, with a split spin–orbit of 23 eV, that could be assigned to Zn metal^31−33^ or ZnO, correspondent to the Zn^2+^ oxidation state.^31−34^ To better distinguish the chemical states of Zn, the principal Zn LMM peak (Auger peak) was collected as it presents larger chemical shifts compared to Zn 2p. The spectra of Zn LMM Auger peaks show the presence of Zn compounds (Figure 4B). The deposited NPs with smaller sizes, TaCaP-Zn1 (Figure 4B, black line) and TaCaP-Zn1C (Figure 4B, red line), only display peaks around 988 eV, which are assigned to ZnO.^35,36^ The presence of ZnO is explained by the higher surface area of the NPs that are easily oxidized on contact with air. The larger NPs, TaCaP-Zn2 (Figure 4B, blue line) and TaCaP-Zn2C (Figure 4B, green line) surfaces, show two compounds. The Zn metal is ascribed to the peak with a kinetic energy of around 992 eV, and the ZnO peak is around 988 eV.^35,36^ The results indicate that the deposited Zn NPs have a core–shell structure of Zn–ZnO NPs caused by the NPs’ surface passivation, as reported by Calderon *et al*.^37^

**Figure 5:**
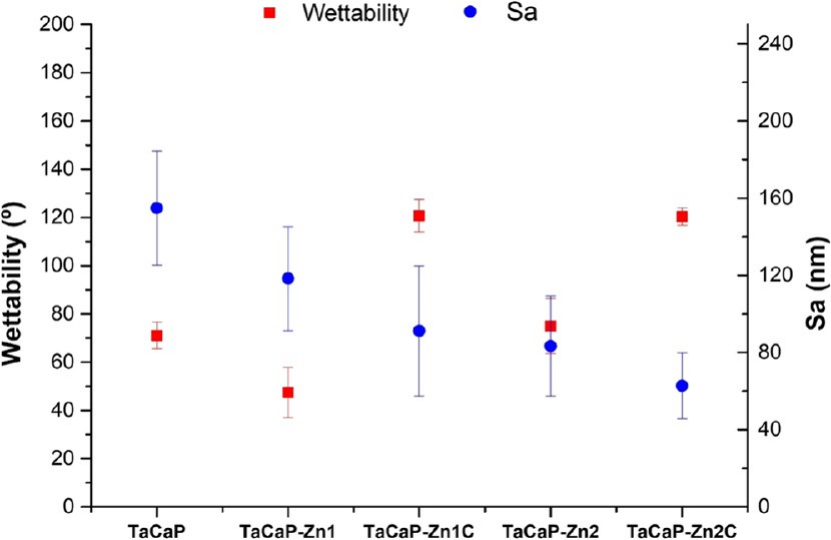
Surface wettability and average roughness (Sa).

The changes induced by the morphological and chemical modification on the TaCaP surface regarding the surface roughness and wettability properties are displayed in Figure 5.

**Figure 6:**
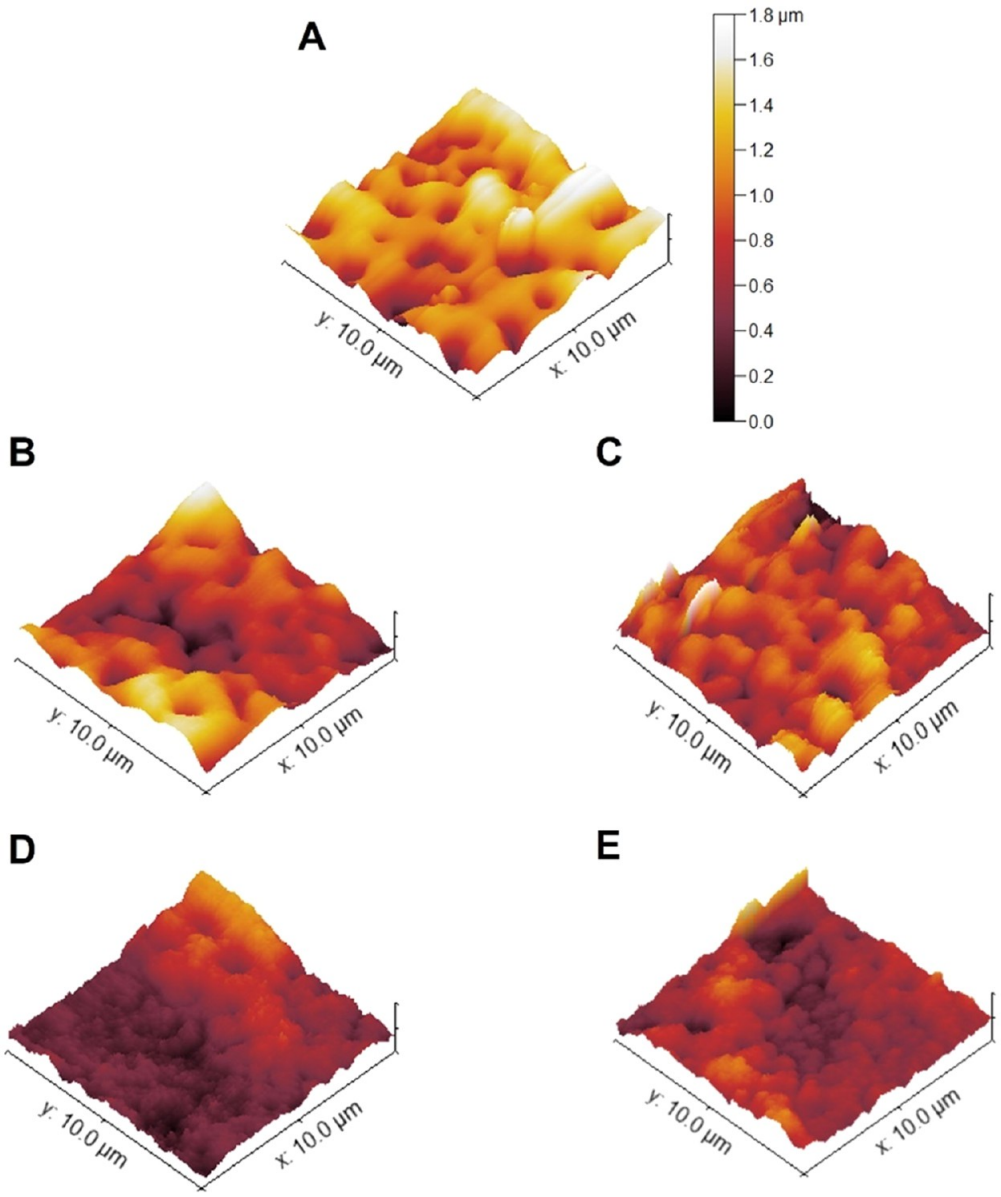
AFM topographic 3D topography images of the (A) TaCaP, (B) TaCaP-Zn1, (C) TaCaP-Zn1C, (D) TaCaP-Zn2, and (E) TaCaP-Zn2C surfaces.

The surface mean roughness (Sa) exhibits a decreasing trend with the Zn NPs and the C layer (Figure 5). This decreased tendency of the surface roughness with the increase of Zn and C content on the surface can be mostly related to the induced changes in the surface morphology. The larger NPs with and without the C layer led to a more significant reduction of the surface roughness as they mitigate the porosity from the micronanostructure (Figure 2D,E), which is less pronounced for smaller NPs (Figure 2B). These topographic modifications induced by the presence of Zn–ZnO NPs with distinct sizes can be observed in Figure 6. The deposition of small Zn–ZnO NPs onto the porous surface changes the surface topography, decreasing the surface roughness (Figure 6B). When larger NPs are deposited with and without the C layer (Figure 6D,E), a cover layer is achieved influencing significantly the surface topography, and the surface becomes smoother, as also noted in Figure 2D,E.

Despite the small differences observed for the samples without carbon, according to Vogler,^38^ the modified surfaces could be considered hydrophobic (water contact angle higher than 60°), except for the TaCaP-Zn1 sample, which could be considered a hydrophilic surface (Figure 5). Surface wettability is ruled by surface roughness and chemical composition. The wetting behavior from the hydrophilic state (TaCaP-Zn1) to the hydrophobic state (TaCaP-Zn2) results from the increase in the content of metallic Zn as well as a decrease in the surface roughness. The presence of the C layer (TaCaP-Zn1C and TaCaP-Zn2C) displays a similar water contact angle, which translates the main role of the C in the surface wettability. These results are in line with the work reported by Lee *et al*.,^39^ who demonstrated that the surface wettability is tailored by the content of ZnO NPs and C. The authors proved that by increasing the deposition cycles of Zn and the C content, the water contact angle can be converted from hydrophilicity to hydrophobicity. Le Dû *et al.*(40) reported that the polyacetylene dissociation is responsible for the hydrophobic character resulting from the CH groups.

### 3.2 Evaluation of the Surfaces’ Antimicrobial Activity

The antimicrobial activity of the surfaces was evaluated through incubation with the pathogenic opportunistic fungus *C. albicans* present in the oral cavity. *C. albicans* can participate in the onset and development of peri-implantitis since its colonization and biofilm formation are usual on metallic implant surfaces.^41^

**Figure 7:**
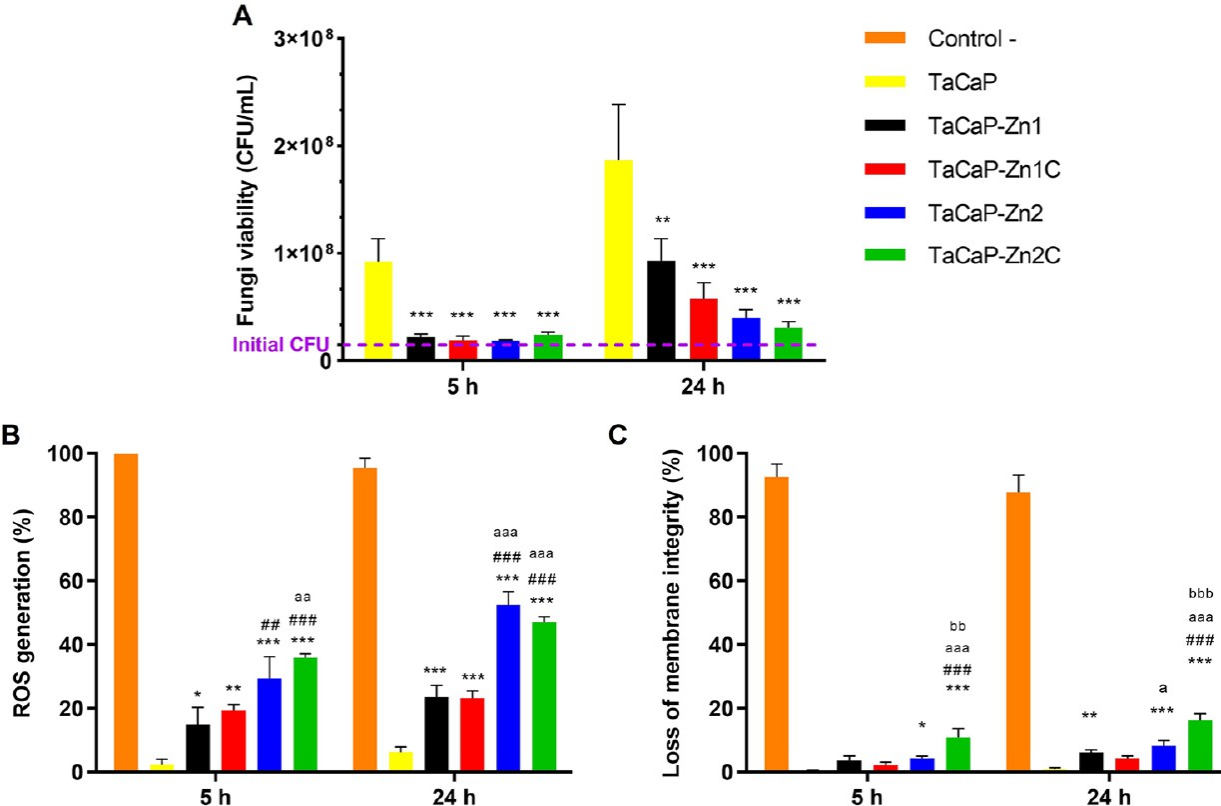
*C. albicans* (A) viability; (B) ROS generation and (C) loss of membrane integrity after 5 and 24 h of culture. Significant values as *p ≤ 0.05, **p ≤ 0.01, and ***p ≤ 0.001, compared to control (TaCaP surface); significant values ^##^*p* ≤ 0.01 and ^###^*p* ≤ 0.001, compared to TaCaP-Zn1; significant values ^aa^*p* ≤ 0.01 and ^aaa^*p* ≤ 0.001, compared to TaCaP-Zn1C; significant value ^bb^*p* ≤ 0.01, compared to TaCaP-Zn2.

The fungi viability was evaluated after 5 and 24 h of contact of the culture with the Zn-containing surfaces, using the TaCaP sample as a control. As seen in Figure 7A, the results reveal a significant reduction in the number of viable fungi in Zn-containing samples, compared to the control (TaCaP) for both time points. After 5 h of contact, the Zn-containing samples disclose similar fungi viability between them and the initial inoculum (Figure 7A, purple dashed line). After 24 h, the fungi viability significantly increases, and it is evident that the number of viable fungi is Zn-dose-dependent, as the TaCaP-Zn2 sample shows a reduction of viable cells compared with TaCaP-Zn1. Although there is no statistically significant difference between the respective surfaces, the samples with the C layer (TaCaP-Zn1C and TaCaP-Zn2C) seem to demonstrate a slight improvement of antimicrobial behavior when compared with their equivalent sample without C coating.

In the literature, it is well reported that ZnO coatings have antimicrobial activity. Pereira-Silva *et al.*(42) studied the antifungal activity of ZnO thin films against*C. albicans* with an inhibition higher than 50 % of viable cell growth. Piedade *et al.*(43) reported that the antibacterial activity of ZnO nanocomposite coatings is significantly improved with the integration of C against *S. aureus* and *Pseudomonas aeruginosa*. ZnO NPs produced by RF magnetron sputtering with a mean size of 20 nm improved the antibacterial effect of the hydroxyapatite substrate as the zone of inhibition growth of *E. coli* bacteria increased,^44^ while ZnO NPs coatings show a minimal effect against *C. albicans*.^45^ Wang *et al*.^17^ produced ZnO NPs to nano-ZnO films onto Ti substrates by RF magnetron sputtering, increasing the deposition time, which increased Zn elemental concentration and the Zn ions released from the surfaces after soaking for 14 days. These ZnO-modified surfaces exhibited an antibacterial effect that occurred in a concentration-dependent manner.^17^ As aforementioned, in our previous work, smaller ZnO NPs (with a maximum level of Zn^2+^ ion release of 0.28 ppm) produced by DC magnetron sputtering led to a decrease of *S. aureus* bacterial viability of around 30%, which is not clinically significant.^14^

Although several mechanisms underlying the antimicrobial activity of ZnO have been reported, the antimicrobial action of ZnO NPs is still under investigation. To understand which mechanisms are responsible for the antifungal behavior, flow cytometry was used to evaluate the ROS generation (Figure 7B) and the cellular membrane integrity (Figure 7C).

The Zn-containing surfaces induce higher ROS formation than the control group (TaCaP) over time, and the surfaces with more Zn–ZnO NPs (TaCaP-Zn2 and TaCaP-Zn2C) exhibit an overwhelming generation of ROS, inducing oxidative stress in about 50% of cells for both time points (Figure 7B). The presence of a C layer does not significantly affect ROS formation when compared to the respective surface without the C coating. The same trend is observed in the loss of membrane integrity (Figure 7C), as the Zn–ZnO NPs induce damage to the *C. albicans* membrane. The TaCaP-Zn2C sample stands out, leading to a loss of membrane integrity of around 20%, which indicates that Zn content is not the only surface property responsible for the antifungal behavior.

In previous work,^24^ the ionic kinetic release from these Zn-containing surfaces was evaluated by ICP-OES for 14 days. The sample with more Zn content releases a higher amount of Zn^2+^ ions throughout the immersion time, whereas the presence of the C coating mitigates the complete release of Zn, which is still encapsulated on the surface. Overall, the amount of Zn^2+^ ions released from each sample tendency is as follows: TaCaP-Zn1C (0.6 ppm) < TaCaP-Zn1 (0.8 ppm) < TaCaP-Zn2C (3.5 ppm) < TaCaP-Zn2 (3.9 ppm).^24^ Taking this into account, the ionic release from TaCaP-Zn1C is sufficient to guarantee a significant inhibition of the fungi growth. El-Belely *et al*.^46^ reported that the ZnO NPs obtained from a green biosynthesis display a minimum inhibitory concentration (MIC) of 12.5 ppm, which is far superior to the ionic release from the porous Ta_2_O_5_ surfaces with Zn–ZnO NPs needed to inhibit the fungi growth (Figure 7). In line with this work, it was observed a concentration-dependent effect of ZnO NPs on antimicrobial activity,^46,47^ as the ROS mediates the cytotoxic effect.^47^ In opposition to this work, Lipovsky *et al*.^47^ noted that the NPs size influences the viability of *C. albicans*. In the present study, the results demonstrate that the Zn–ZnO NP-containing surfaces have a clear impact in inhibiting fungi adhesion and proliferation and that effect is dose-dependent. The main mechanisms of action of the Zn–ZnO NPs to decrease fungi viability seem to be the ionic kinetic release and the oxidative stress generation (Figure 7B) that impaired cell damage repair and survival, while the loss of the membrane integrity had a minor effect (Figure 7C). No size dependence of Zn–ZnO NPs was observed.

In line with this work, it was observed a concentration-dependent effect of ZnO NPs on antimicrobial activity,^46,47^ as the ROS mediates the cytotoxic effect.^47^

**Figure 8:**
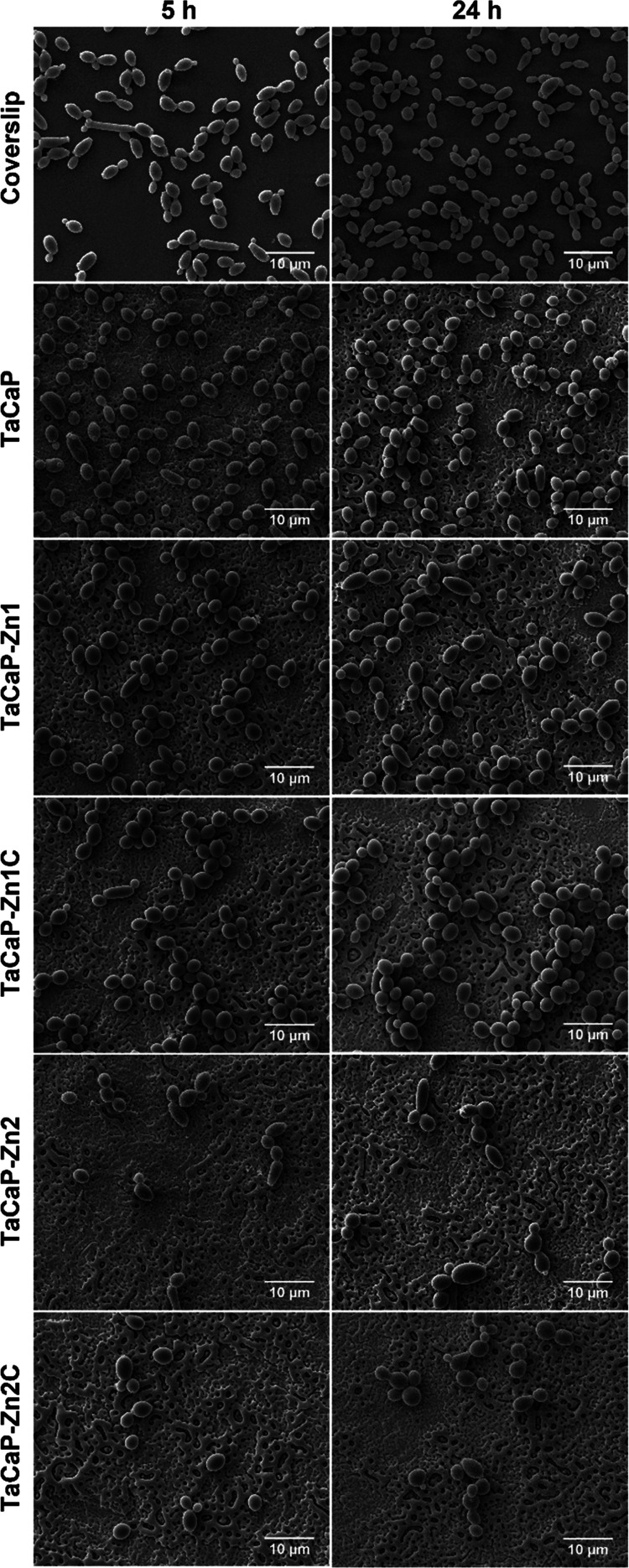
SEM micrographs of *C. albicans* adherence on the coverslip (used as the fungi morphological control) and modified surfaces after 5 and 24 h of culture. Scale bar: 10 μm.

The fungi morphology on the samples’ surfaces, displayed in Figure 8 shows a difference between the Ta-modified surfaces and the glass coverslip control. The fungi on the control presented the known *C. albicans*different morphologies (yeast and pseudohyphae^48^), as the pseudohyphae morphology is longer in the control. It is also evident that the quantity of adherent fungi to the surface is Zn-dose-dependent. Compared with the CFU results (Figure 7A), it is evident that the Zn–ZnO NPs induce a fungistatic behavior and that some of the cells that adhere to the surface will lose viability.

As it is well known, the chemical composition and the surface morphology have a strong influence on microbial responses. Thus, a possible explanation for these results may be the fact that the C layer leads to partial mitigation of the pores at the nanoscale, which are preferential spots for cell adhesion, decreasing the roughness and leading to a hydrophobic surface, thus making it more difficult for *C. albicans* to adhere and proliferate (Figure 8).

### 3.3 Cytotoxicity and Inflammatory Responses

**Figure 9:**
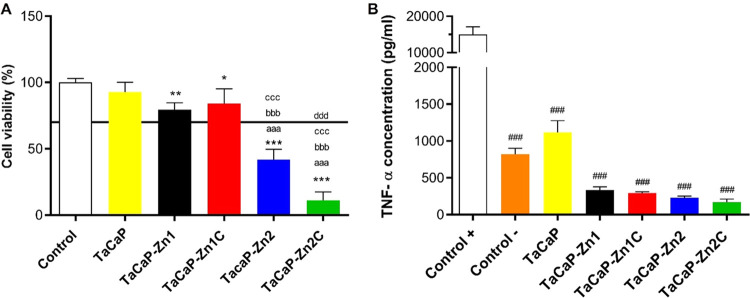
(A) Macrophages’ metabolic activity and (B) TNF-α concentration after 24 h of incubation. Control indicates macrophages grown on plastic cell culture plates; Control + indicates LPS-stimulated macrophages and Control – nonstimulated macrophages. Significant values as *p ≤ 0.001, compared to the control; significant values as ^###^*p* ≤ 0.001, compared to the control +; significant values as ^aaa^*p* ≤ 0.001, compared to the TaCaP; significant values as ^bbb^*p* ≤ 0.001, compared to the TaCaP-Zn1; significant values as ^ccc^*p* ≤ 0.001, compared to the TaCaP-Zn1C; significant values as ^ddd^*p* ≤ 0.001, compared to the TaCaP-Zn2.

Before analyzing macrophage response to the different surfaces, it is important to determine their cell toxicity. After 24 h of incubation, macrophages on TaCaP samples are metabolically active similar to the control group of macrophages grown on plastic cell culture plates (Figure 9A). This result is in line with the literature that indicates the micro/nanoporosity of the Ta_2_O_5_ surface enriched with CaP increases the osteoblastic cells’ adhesion and proliferation.^14^ The metabolic activity of the macrophages on the porous Ta_2_O_5_ surfaces doped with the smaller Zn NPs (TaCaP-Zn1 and TaCaP-Zn1C) is similar to that on the TaCaP surface, whereas a significant decrease is observed on TaCaP-Zn2. This behavior means that the presence of larger Zn–ZnO NPs compromises the macrophages’ activity, mostly when the NPs are covered by the C layer (TaCaP-Zn2C), as both surfaces show a cytotoxic effect, as an effect of a smoother and hydrophobic surface. These results reveal that there is a threshold of Zn content that enhances microbial cellular adhesion and proliferation and can be used safely in animal cells. According to the literature, the Zn ion becomes cytotoxic when it reaches a release concentration of 10 ppm.^49^ So, there is a Zn-dose dependence that can cause macrophage dysfunction and ultimately macrophage death. Most of the literature reports a size-dependent cytotoxic profile, as smaller NPs display greater effect,^50,51^ and lower ZnO NP concentration and higher Zn^2+^ release are associated with higher toxicity in macrophages.^50^ IIn this study, CaP-enriched porous Ta2O5 surfaces with the Zn-ZnO NPs show a dose-dependent effect on cellular viability, as the highest NPs concentration and the highest ionic release lead to a greater toxic effect. The size of NPs does not disclose a significant impact on macrophage viability.

To evaluate the inflammatory response upon incubation with the modified Ta_2_O_5_ surfaces, the cytokines TNF-α (proinflammatory) and IL-10 (anti-inflammatory)^10^ were quantified.

As seen in Figure 9B, the roughest TaCaP surface shows a higher TNF-α level than untreated cells (control −), while the deposition of Zn–ZnO NPs did not enhance TNF-α production; on the opposite, it significantly reduced this proinflammatory cytokine. These results suggest that the TNF-α cytokine release is sensible to the surface chemistry, namely, to the presence of Zn–ZnO NPs, and the roughness of the surface. None of the modified surfaces cause IL-10 cytokine release. The herein results (Figure 9B) show that there is a significant suppression of the inflammatory state induced by the modified surfaces compared to the LPS-stimulated cells (control +), which means that the modified surfaces do not induce an inflammatory response from the macrophages and decrease the possibility of implant aseptic loosing.^1^ TNF-α is a strong proinflammatory cytokine and one of the most abundant early mediators in inflamed tissues, being mostly produced by cells of the monocyte lineage.^52^ Thus, a slight TNF-α production from macrophages can lead to the cell recruitment and antifungal action of macrophages and other inflammatory cells without severe inflammation.^53^ The results are in agreement with the literature. Nagajyothi et al.^54^ demonstrated a dose-dependent suppression of both the mRNA and protein expressions of TNF-α by ZnO NPs. Nano-ZnO films promote the secretion of inflammatory cytokines from macrophages after LPS stimulation, such as TNF- α, in a dose-dependent manner.^17^

**Figure 10:**
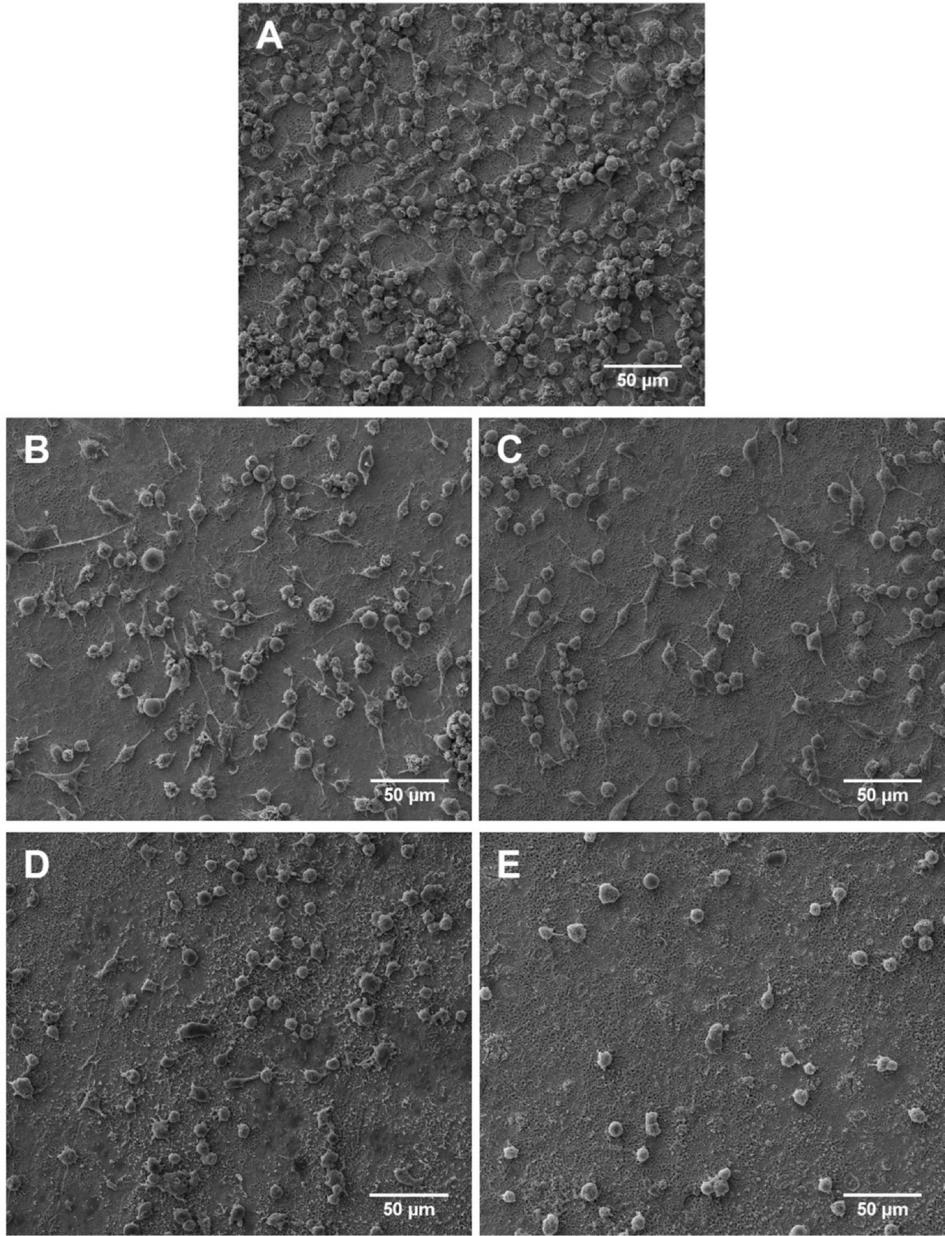
SEM micrographs of macrophage cell morphology after adhesion following 24 h of incubation on (A) TaCaP, (B) TaCaP-Zn1, (C) TaCaP-Zn1C, (D) TaCaP-Zn2, and (E) TaCaP-Zn2C surfaces. Scale bar: 50 μm.

Macrophage morphology after 24 h of incubation on the porous TaCaP surface shows cells densely packed with a rounded shape and pronounced cytoplasmatic extensions (Figure 10A). For both TaCaP-Zn1 (Figure 10B) and TaCaP-Zn1C (Figure 10C), cells are more dispersed along the surface, with no evidence of clusters. Macrophages display a rounded and elongated morphology and cytoplasmatic extensions. For both TaCaP-Zn2 (Figure 10D) and TaCaP-Zn2C (Figure 10E), macrophages have predominantly a rounded shape with weaker and thinner cytoplasmatic extensions. In line with the macrophage’s cell viability (Figure 9A), the TaCaP surface is preferential to cell adhesion and proliferation than Zn-containing surfaces, which suggests that macrophage adhesion and proliferation are improved by the rougher surface and are also Zn-dose-dependent.

The presence of smaller Zn NPs (TaCaP-Zn1), hydrophilic and rough surface, leads to an inhibition of fungi viability with no toxicity to macrophages. When the thin C layer is deposited over the smaller Zn NPs (TaCaP-Zn1C), a super hydrophobic and moderated rough surface leads to a more significant fungistatic effect and superior macrophage viability. When larger Zn NPs are deposited (TaCaP-Zn2), hydrophobic and smooth surface, a more pronounced inhibition of *C. albicans* and macrophage toxicity is observed, being more significant with the presence of the C layer (TaCaP-Zn2C), the super hydrophobic and smoother surface. Thus, the surface wettability (Figure 5) appears to influence neither the fungi nor macrophage’s viability of the surfaces, as the hydrophilic surface (TaCaP-Zn1) does not show different cellular behavior compared to the other hydrophobic surfaces (TaCaP-Zn1C). Also, surface roughness (Figure 5), by itself, shows an evident relation to both fungi (Figure 7A) and macrophages (Figure 9A) viability. Yet, the roughest TaCaP surface leads to the high expression of proinflammatory cytokines (Figure 9B), as reported in the literature,^10^ as well as leads to a low ROS generation (Figure 7B) without affecting the fungi’s membrane integrity (Figure 7C). Among the discussed surface properties, surface chemistry (Zn-dose-dependent behavior combined with the presence of the C layer) seems to have the main effect on antifungal behavior and immune activation. Taking the discussed results into account, the TaCaP-Zn1C surface sample is the one that allows simultaneously a fungistatic behavior (Figures 7A and 8), possibly due to the ionic kinetic release^24^ and/or ROS generation (Figure 7B), and macrophage cell viability (Figures 9A and 10), suppressing the proinflammatory cytokine expression (Figure 9B). Our previous results^14^ demonstrated the ability of rougher porous Ta_2_O_5_ surfaces with smaller Zn–ZnO NPs coated with the thin C layer to promote the initial adhesion and proliferation of osteoblastic cells. These previous results in combination with this study show promising evidence for the benefits of porous Ta_2_O_5_ surfaces with Zn–ZnO NPs coated with the thin C in dental implantology.

## 4 Conclusions

The deposition of Zn–ZnO NPs on porous CaP-enriched Ta_2_O_5_ surfaces was performed by DC magnetron sputtering, a green and well-industrialized technique that allows the production of a large batch of samples during a single deposition process. The size and amount-dependent Zn were investigated to improve the antifungal activity of endosseous implants without inducing an inflammatory response. *C. albicans* viability was revealed to be Zn-dose-dependent, as the surfaces with the highest amount of Zn–ZnO NPs displayed the highest decrease in fungi viability because they lead to higher ROS generation and Zn^2+^ release. It was observed that there is a Zn amount threshold between the antifungal behavior and cytotoxicity, and the TaCaP-Zn1C surface sample translates the best compromise between antifungal and cytotoxicity. Thus, the surface chemistry, i.e., the Zn content and the presence of the C layer, appears to have an overwhelming antifungal and immunomodulatory effect. To guarantee the effectiveness of these bioactive surfaces, further studies should be performed to assess their antimicrobial and immunomodulatory effects against various oral microorganisms when co-cultured with macrophages. Additionally, exploring the potential of designing a multilayer coating of C with the Zn–ZnO NPs would be interesting, since this approach has the potential to regulate sustained release of the antimicrobial agent over an extended period.
